# The concept of disease in the time of COVID-19

**DOI:** 10.1007/s11017-021-09540-5

**Published:** 2021-02-16

**Authors:** Maria Cristina Amoretti, Elisabetta Lalumera

**Affiliations:** 1 DAFIST, Philosophy Section, University of Genoa, Via Balbi 4, 16126 Genoa, Italy; 2 PhilHeaD, Research Center for Philosophy of Health and Disease, Genoa, Italy; 3 Department for Life Quality Studies, University of Bologna, Largo Augusto 230, 47921 Rimini, Italy

**Keywords:** COVID-19, Concept of disease, Harm, Pandemic, Sick role

## Abstract

Philosophers of medicine have formulated different accounts of the concept of disease. Which concept of disease one assumes has implications for what conditions count as diseases and, by extension, who may be regarded as having a disease (disease judgements) and for who may be accorded the social privileges and personal responsibilities associated with being sick (sickness judgements). In this article, we consider an ideal diagnostic test for coronavirus disease 2019 (COVID-19) infection with respect to four groups of people—positive and asymptomatic; positive and symptomatic; negative; and untested—and show how different concepts of disease impact on the disease and sickness judgements for these groups. The suggestion is that sickness judgements and social measures akin to those experienced during the current COVID-19 outbreak presuppose a concept of disease containing social (risk of) harm as a component. We indicate the problems that arise when adopting this kind of disease concept beyond a state of emergency.

## Introduction

Philosophers of medicine have formulated different accounts of the concept of disease [1–5].^1^ Some identify dysfunction as a requirement for disease [6, 7, 14–17]; others claim that a condition needs to cause harm, be undesirable, or put one at risk in order to be a disease [8–11, 18–20]. The view that both dysfunction and harm are components of the concept of disease has also been defended [5, 13, 21, 22].

Which concept of disease one assumes has implications for what counts as a disease (nosology). In at least one case, this implication was enforced by an explicit stipulation on the part of the scientific community. In 1981, the third edition of the *Diagnostic and Statistical Manual of Mental Disorders* (*DSM*) contained a definition of mental disorder that included a harm requirement (necessitating distress or disability to the individual) so that homosexuality could be coherently eliminated from the catalogue of diseases [23]. This move changed the applicability of what we call ‘disease judgements’, or judgements about what conditions count as diseases and, by extension, who can be regarded as having a disease, in psychiatry: given that homosexuality does not cause harm and is therefore not a disease according to the current definition of mental disorder, people who are homosexual cannot be regarded as having a disease.

Concepts of disease also have implications for what we call ‘sickness judgements’, or judgements about how the rights and restrictions associated with forms of sickness are attributed to individuals by virtue of their condition (e.g., leave from work, benefits, entitlement to treatment and reimbursements, or the obligation to surrender one’s driving license). Sickness is the social aspect of disease. While disease and sickness judgements do not always correspond, the concept of disease puts constraints on what counts as sickness [24–27].

In the classical tradition of conceptual analysis, philosophical discussions about concepts of disease consist of confronting a proposed account with a certain condition (e.g., a dysfunction-requiring account with hypercholesterolemia, given a certain definition of dysfunction), assessing whether the concept applies to that condition, and then evaluating whether the verdict matches one’s intuitions [28–30]. Here, our methodology is different. We will run a simple thought experiment considering an ideal diagnostic test for an infectious disease, such as coronavirus disease 2019 (COVID-19), with respect to four groups of people—positive and asymptomatic; positive and symptomatic; negative; and untested—and show how different concepts of disease produce distinctive disease and sickness judgements for these groups. Thus, assuming disease judgements map to disease concepts, we can infer what concept is implicit in particular situations by looking to the associated judgement patterns. Accordingly, we conclude that the pattern of sickness judgements seen during the COVID-19 outbreak in many countries underlies a concept of disease as social (risk of) harm. We then point to some of the problems that arise when adopting this kind of disease concept beyond a state of emergency. In so doing, however, our goal is neither to offer a positive concept of disease to be used by authorities nor to criticize the various policies and measures that governments have actually implemented during the pandemic.

The structure of the paper is as follows. We begin in the following section by clarifying our terminology—specifically, our use of the terms ‘disease judgement’ and ‘sickness judgement’. We then run our thought experiment and summarize its results in the second section. In the third section, we argue that the sickness judgement patterns found in many nations during pandemics are consistent with a concept of disease that contains a component of social (risk of) harm, and we highlight the undesirable consequences of endorsing such a concept beyond exigent circumstances of emergency. We conclude by considering two objections to our argument.

## Preliminary issues

Adopting any concept of disease commits and entitles one to hold that some conditions are diseases and others are not. For example, conceptualizing disease as dysfunction—defined either as deviation from normality relative to a reference class or as deviation of a part from its species-typical contribution to survival and reproduction—commits one to the view that the death of a single neuron is a disease and ‘a normal person is anyone who has not been sufficiently investigated’ [7, p. 49] (quoting [31, p. 123]). Similarly, in assuming a concept of disease that includes individual risk as a requirement, people who are obese are counted as diseased whereas people with very small asymptomatic cancers are not [32].

Conversely, to maintain that some condition is (or is not) a disease, one may have to propose or defend a new disease concept. As mentioned above, its espousal of a harmful dysfunction view of mental disorder allowed the American Psychiatric Association to avoid regarding people who are homosexual as having a disease. Likewise, it may be expedient to grant the status of disease to unwanted pregnancy, in which case a concept of disease as harm to the individual is required [8, pp. 278–279]. Less controversially, granting long-term treatment and therapies to people with high cholesterol levels requires a concept of disease that includes a component of individual risk.

We use ‘disease judgements’ in reference to those judgements that categorize a given condition as a disease and, by extension, a given group or individual who has that condition as diseased. Sometimes disease judgements may vary from person to person. For example, following an account of disease that has harm to the individual as a component, labial herpes would count as a disease when symptoms are present, but not when it is asymptomatic as there is no present harm to the individual. Consequently, we think it is necessary to consider symptomaticity as a variable in the thought experiment we run in the next section.

The relation between disease judgements and concepts of diseases is logical, as they imply each other [33, 34]. However, from an epistemic point of view, one can proceed in two ways: either by proposing a new concept of disease in order to license or block certain disease judgements (as in the examples above) or by analysing the disease judgements implicit in some situations and distilling or making explicit which disease concepts correspond to them. In this paper, we take the latter course, illustrating the commitments of different concepts vis-à-vis our imaginary but realistic case and then criticizing the concept that best describes a certain set of judgement patterns.

We are aware that the phrase ‘disease judgement’ is somewhat awkward, but we need to keep the notion separate from clinical judgement and diagnosis. While a disease judgement just categorizes a condition or group of people as being or having a disease respectively, clinical judgement and diagnosis stand for more complex processes or events and concern individual patients. Clinical judgement ‘refers to the range of complex reasoning tasks and actions performed by clinicians in the context of offering diagnosis, therapeutic options, and prognosis to patients’ [35, p. 363] (see also [36, p. xxii]). In simple terms, a clinical judgement or diagnosis (as an event) involves identification of some condition or conditions that explain the signs or symptoms of a given patient, typically through use of a standardized test or personal examination (see, e.g., [37]). Here, the word ‘condition’ is used to capture the fact that not all clinical judgements attribute diseases to people, as illustrated intuitively by a normal pregnancy case.

By ‘sickness judgement’ we mean the attribution of a sick role (consisting in privileges and restrictions) to individuals by virtue of their condition, as mentioned above. The notion of sickness as the social dimension of disease comes from sociology [26, 27] and it is often invoked in the context of the disease–illness–sickness triad—where disease is an objectively detectable condition and illness is the subjective unease experienced by one who has a condition [24, 25].

From a descriptive point of view, sickness judgements are not isomorphic to disease judgements. As Bjørn Hofmann observes, conditions like fibromyalgia, low back pain, whiplash, and chronic fatigue syndrome may entitle one to be considered sick by society but are not diseases according to the medical profession; and chronic conditions like hyperglycaemia, hypertension, and food/pollen allergies are still diseases when asymptomatic but do not warrant a sick role [24, p. 658]. Nevertheless, from a normative point of view, disease concepts and therefore disease judgements are often criticized or revised precisely in order to block or permit sickness judgements for some categories of people. This was the case for homosexuality, as mentioned above, as well as for drapetomania, a disease proposed in the mid-nineteenth century to diagnose slaves who had an uncontrollable or insane impulse to run away from servitude [38]. In fact, this situation is frequent in psychiatry and clinical psychology, where there is arguably less consensus on nosology in comparison to other branches of medicine. Rachel Cooper [39] and Miriam Solomon [40] observe that when a certain condition is included in the *DSM*, people who suffer from it are entitled to therapies and reimbursements (see also [41]). In general, philosophical theories of disease put important constraints on sickness judgements insofar as sickness judgements are taken either to coincide with disease judgements or to be contingent on one’s already having received a disease judgement.

Above we mentioned the disease–illness–sickness triad. However, we do not systematically consider ‘illness judgements’, that is, those judgements by which someone is recognized and described as suffering from or distressed by a condition, either by oneself or by another person. This, of course, does not mean that illness judgements are irrelevant, just that their analysis is not directly connected to our primary goal—determining what concept of disease lies behind sickness judgements akin to those observed during the COVID-19 outbreak in many countries.

## Assessing concepts with an imaginary diagnostic test

We are now ready to introduce our thought experiment. Let us consider a diagnostic test for the severe acute respiratory syndrome coronavirus 2 (SARS-CoV-2) infection that causes COVID-19 [42]. Note that this test is an ideal one: we assume that a consensus has been reached on a test that is optimally accurate (i.e., both specific and sensitive), meaning that false positive and false negative ratios are zero.^2^ In reality, according to current diagnostic guidelines at the time of writing, the standard procedure for diagnosing COVID-19 involves laboratory-based swab tests for viral nucleic acid detection [43]. However, although there is evidence for the specificity of such testing, sensitivity appears to be sub-optimal and other tests are currently being evaluated and proposed [44]. In our thought experiment, we abstract away from these problems.

Now let us consider a group of people (we could use the term ‘sample’ here, but it would be in a non-technical sense), focusing on COVID-19 only and assuming that no other disease is present. With respect to our ideal diagnostic test for COVID-19, four classes can be identified: positive and symptomatic (PS), positive and asymptomatic (PA), negative (N), and untested (U). We assume that the PS group comprises people with more or less severe symptoms and that the PA group comprises people with mild symptoms or no symptoms at all. We also assume that the N group includes people who have never encountered the infection and people who have recovered, whereas the U group may contain both infected (symptomatic or asymptomatic) and noninfected people. In discussing the various concepts of disease, we will make these specifications explicit where relevant.

Which of the four classes contain the people diseased with COVID-19? It is not clear that ‘PS and PA’ is the answer. Obviously COVID-19 *is* a disease—in fact, it is a terrible disease that has caused a worldwide pandemic according to WHO [45]. What is not obvious is how different philosophical accounts of disease would characterize individuals within the four classes—or alternatively, which concept of disease is implicit in certain patterns of disease and sickness judgements. This is what we aim to investigate.^3^

Christopher Boorse developed the most prominent naturalistic account of health and disease [6, 7, 14–16], dubbed the biostatistical theory (BST) because it rests on concepts of biological function and statistical normality with the aim of delineating a value-free scientific definition of health and disease. According to the BST, disease—broadly conceived to include all pathological conditions—is a type of internal state that is either an impairment of normal functional ability (i.e., a reduction of functional abilities below typical efficiency) or a limitation on functional ability caused by environmental agents [6, pp. 562, 566]. Typical efficiency, namely, the statistically typical contribution by a part or process within an organism to its survival or reproduction, must be determined in relation to a reference class, namely, an age group of a sex of a species. Moreover, Boorse also draws an important distinction between disease, which is a primary theoretical concept, and secondary value-laden ‘disease-plus’ concepts: ‘Starting from the basic disease concept, one can define clinically evident disease, or harmful disease, or serious disease, or treatable disease, or disabling disease, or disease that should be covered by insurance, or disease that should remove civil or criminal responsibility, and so on’ [7, p. 100]. Sickness judgements are similarly secondary with respect to disease judgements in that no one can receive a sickness judgement without having first received a disease judgement.

From a Boorsian position, people in the PS group would count as diseased. Depending on the severity of symptoms and the cultural and social values at play, they would probably be accorded the sick role too. Therefore, the PS group would receive a positive disease judgement, while individual sickness judgements would likely be positive but may vary with symptom severity and against different backdrops of cultural understandings and social norms. People in the PA group would also count as diseased, but given the absence or triviality of symptoms, they may not be granted the sick role (again, this would depend on the social values at play). So the PA group would have a positive disease judgement and probably a negative sickness judgement. A tested and negative subject is simply not diseased. Since, in Boorse’s view, sickness judgements depend on disease judgements, both judgements would be negative for the N group. The situation is different for people in the U group, each of whom in principle may or may not be diseased. As long as no test has been conducted, however, no disease judgement can be rendered, and thus a negative disease judgement is the default. The U group’s sickness judgement would also be negative, then, at least as long as no test has been performed and no positive disease judgement has been rendered.

Another account that endorses the dysfunction requirement as objective and value-free, but combines it with a normative judgement, is the harmful dysfunction theory of disease developed by Jerome Wakefield [13, 22].^4^ For Wakefield, a condition is a disease if and only if it results from the inability of some internal mechanism to perform a particular function that forms part of the evolutionary explanation for the existence and structure of that mechanism (dysfunction),^5^ and the condition impinges harmfully on the subject, as judged by the social values and meanings shared by the subject’s community and culture (harm) [13]. On this view, any disease must underlie a dysfunction, and that dysfunction must also cause some individual harm in present environmental circumstances and according to present cultural standards. Put differently, only dysfunctions that cause some harm or deprivation of benefit to the subject, as judged by the standards of the subject’s culture and social values, may be considered diseases. According to Wakefield, the harmful dysfunction account played a key role in blocking positive sickness judgements for homosexual people [46], and it is also pivotal in preventing false positives in psychiatry (in which a diagnosis of mental disorder is made for non-disordered people) [22]. In this sense, disease judgements and sickness judgements are strictly linked and tend to coincide.

Adopting Wakefield’s concept, people in the PS group would count as diseased: there is a dysfunction and that dysfunction is socially judged to have a harmful impact on the subject (recall that we are assuming that symptoms in this group are relatively severe). As such, the disease judgement as well as the sickness judgement would be positive. People in the PA group certainly have a dysfunction, but they may or may not count as diseased depending on how the condition is evaluated by present cultural standards. For example, by contemporary Western cultural standards, an asymptomatic cancer patient would count as diseased insofar as the dysfunction is judged to be harmful to the subject, whereas a person with one kidney would not count as diseased insofar having one kidney is often seen to have no impact on one’s overall well-being and so the dysfunction is not socially judged to be harmful to the subject [13, p. 384]. In the case of COVID-19, people in the PA group would have a dysfunction that is probably socially judged not to have a significant harmful impact on them; hence they would not count as diseased, and their disease and sickness judgements would be negative. A tested and negative subject is not diseased as no dysfunction is present; again, the disease and sickness judgements for the N group would be negative. It is not known whether or not a dysfunction is present in the U group, so it would be important to establish the occurrence of potential symptoms and evaluate their pathosuggestiveness—that is, the extent to which they support an inference to underlying dysfunction [47]. A subject who displays sufficiently pathosuggestive symptoms, such as pneumonia or high fever, would be regarded as diseased; conversely, a subject who displays only minor symptoms, such as fatigue or mild fever, would not be regarded as diseased. However, even assuming the presence of sufficiently pathosuggestive symptoms, since similar symptoms might be consistent with different kinds of disease and a diagnostic test is feasible to reliably identify COVID-19, disease and sickness judgements with respect to COVID-19 should be negative until the diagnostic test is actually performed.

We will move now to a wholly normative account of disease, proposed by Danner Clouser, Charles Culver, and Bernard Gert [10, 11].^6^ According to Clouser et al., a disease is an internal condition outside of one’s rational beliefs or desires that has no distinct sustaining cause,^7^ by virtue of which the subject incurs or is at significantly increased risk of incurring a basic harm (i.e., death, pain, disability, loss of freedom or opportunity, or loss of pleasure).^8^ A viral infection can, in theory, count as a disease in this sense: it is an internal condition, it is not a rational belief or desire, it has no distinct sustaining cause once the virus is biologically integrated into one’s body, and it often involves incurring harm or a significantly increased risk of incurring harm. However, whether or not such a condition counts as a disease depends on the actual level of harm incurred or extent to which one’s risk of harm is increased. It is important to stress that level of harm, or risk of harm, must always be evaluated in relation to the subject at hand. As with prior accounts, disease judgements are strictly linked to sickness judgements in that the former can be defended or criticized in order to block or permit the latter.

With the disease concept advanced by Clouser et al., people in the PS group would count as diseased, yielding a positive disease judgement, because they have an internal condition that involves not only the current incurring of harm (e.g., pain), but also, at least for some groups, a significantly increased risk of incurring harm (e.g., death). If harm or potential harm is significant enough, the group’s sickness judgement would be positive as well. People in the PA group are not currently experiencing significant harm. Of course, there are many conditions, such as HIV or hypertension, that may not incur harm initially but will definitely or very likely precipitate harm in the (near) future; for Clouser et al., these conditions are also regarded as diseases in view of the significantly increased risk of incurring harm [11]. In the case of COVID-19, the increased risk of incurring harm may vary depending on the characteristics of the group in question; the increased risk of incurring harm may be minor for certain groups, such as young people without cooccurring pathologies, and significant for others, such as the elderly and people who are immunosuppressed. In the absence of present harm, then, the former would count as healthy and the latter as diseased. Disease and sickness judgements for the PA group would distribute accordingly: they would be positive only for those with significantly increased risk, such as the elderly and people who are immunosuppressed, and negative for everyone else. By contrast, tested and negative subjects would not count as diseased because no viral infection is present. To be considered diseased, it is not sufficient that a subject belong to an at-risk category of some sort, such as the elderly; rather, an identifiable internal condition (i.e., viral infection) must be associated with the subject’s predicted significant harm [12]. The disease and sickness judgements for the N group would therefore both be negative. An untested patient theoretically may or may not meet the requirements for having a disease under this definition. However, as long as no internal condition has actually been identified using a diagnostic test, no disease judgement with regard to COVID-19 can be rendered for the U group, making their sickness judgement negative as well.

According to Rachel Cooper, the term ‘disease’ serves to pick out those conditions whose harmfulness makes them of interest to us as people [8, 9]. More precisely, her tripartite analysis defines a disease as a condition (i) that is a bad thing to have, (ii) that is such that one would consider the afflicted person to have been unlucky, and (iii) that can potentially be medically treated [8, p. 279]. The first criterion works to distinguish diseases from mere biological differences (such as ginger hair). It is worth noting that for Cooper the subject herself has to assess whether or not a certain condition, evaluated in and of itself, is bad (or harmful); in theory, then, the same condition may be a bad thing for one person but a good thing for another. The second requirement, establishing that a diseased subject could reasonably have hoped to have been otherwise, works to distinguish diseases from harmful but normal conditions (such as teething). Finally, the third requirement works to distinguish diseases from other misfortunes (such as poverty). Here too disease and sickness judgements are taken to be strictly connected.

Applying Cooper’s disease concept, people in the PS group may be seen to have a condition that is such that one would consider an afflicted person to have been unlucky and that is potentially medically treatable. That being said, it is not straightforward that the condition is necessarily bad for an individual subject, who alone is entitled to evaluate its badness. According to Cooper, ‘in the vast majority of cases there will be no disagreement between people as to whether or not a condition is a bad thing’ in and of itself, especially if symptoms are severe [8, p. 275]. Presumably, most people in the group would consider COVID-19 a bad thing to have, and so would count as diseased. Still, it is technically possible that someone does disagree; in that case, the subject would not count as diseased. Thus, we can say that disease and sickness judgements would be positive for the majority of PS people, but there might be some special situations in which these judgements would be negative. Much like people in the PS group, people in the PA group may also be seen to have a condition that is such that one would consider an afflicted person to have been unlucky and that is potentially medically treatable. Here, however, it may be reasonable to suppose that most people in the group would not consider asymptomatic COVID-19 to be a bad thing to have in and of itself, and so would not count as diseased. Again, it is possible that someone does indeed consider this a bad thing to have, and that subject would count as diseased. Mirroring the previous group, then, we can say that disease and sickness judgements would be negative for the majority of PA people, but there might be some special situations in which these judgements would be positive. A negative subject would not be considered diseased, as none of the three criteria of the disease definition is satisfied. So the disease and sickness judgements for the N group would be negative. Whether or not people in the U group would count as diseased likely depends on the COVID-19 symptoms they are experiencing: those whose COVID-19 symptoms are sufficiently severe to satisfy the three criteria of the disease definition would count as diseased; those without symptoms or whose symptoms are minor or mild would not count as diseased. Their disease and sickness judgements would distribute accordingly.

In all of the above definitions of disease, discussion of harm (or increased risk of harm) refers explicitly to harm (or increased risk of harm) to the subject. Prima facie, it seems reasonable to suppose that the diseased subject is the one who is harmed (or at an increased risk of being harmed) by the pathological condition. However, it is possible to define disease in terms of harm (or increased risk of harm) to someone other than the subject. Specifically, one could say that a condition is a disease if it is harmful to specific groups of people or to society. Implicitly, this position is partly endorsed by the *DSM*’s definition of mental disorder, according to which a mental disorder is a syndrome that ‘reflects a dysfunction’ and is ‘usually associated with significant distress or disability’ [48, p. 20].^9^ At least some mental disorders—such as pyromania, kleptomania, antisocial personality disorder, and paedophilia—are considered to be pathological conditions because they are dysfunctions that cause harm or increased risk of harm to specific groups of people or society as a whole [50, 51]. As we argue elsewhere, for each of these disorders, ‘the harm potentially experienced by the subject (e.g., via imprisonment, isolation from the community, and so forth) seems to be not only indirect—stemming not from the underlying dysfunction itself, but from the society the subject lives in—but also irrelevant to the diagnosis. In fact, the harm to assess seems to be that experienced by people other than the disordered subject’ [50, p. 333]. For example, paedophilia is considered to be a mental disorder because it is a dysfunction that ‘results in the victimization of children’ [51, p. 431]—that is, because it is harmful to children, not to the subject. Similarly, antisocial personality disorder is considered to be a mental disorder because it is characterized by ‘a pervasive pattern of disregard for and violation of the rights of others’ [49, p. 659]—that is, because it causes harm to people other than the subject [50, p. 333]. In view of such considerations, disease could be explicitly defined as a condition that is usually associated with some harm, or increased risk of harm, either to the subject or to someone other than the subject (specific groups of people or society as a whole).^10^ Here too disease and sickness judgements can be regarded as strictly connected.

By this definition, which we can call the social (risk of) harm account, people in the PS group would count as diseased: they have a condition that is not only currently harmful to them, but also potentially harmful to society, or at least to certain groups of at-risk people (such as the elderly). People in the PA group would also count as diseased: the condition, though not currently harmful to them, can still increase the risk of harm to them and, more relevantly, is also potentially harmful to society, or at least to certain at-risk groups therein. For both groups, then, disease and sickness judgements would be positive. By contrast, people in the N group are not diseased as negative subjects have no condition that may cause harm (to the subject or to others); hence their disease and sickness judgements would be negative. Finally, the U group may in principle contain noninfected people and infected people, whether symptomatic or asymptomatic. Of course, regardless of one’s symptoms, without performing a test it is not possible to rule out the presence of the virus, which may in turn infect other people. The mere fact of being untested thus represents an increased risk of harm to society, especially to at-risk groups (though it may not represent an increased risk of harm to the subject, at least in the absence of symptoms). If disease is defined as a condition that is usually associated with some harm, or increased risk of harm, either to the subject or to someone other than the subject, then all untested people must be considered diseased. The U group’s disease and sickness judgements would therefore be positive.

In summary (see Table 1), on all of the above accounts of disease (with the possible exception of Cooper’s theory), people in the PS group count as diseased and people in the N group do not count as diseased; hence disease and sickness judgements would be positive for the former and negative for the latter. With respect to the PA group: the BST would engender a positive disease judgement but likely a negative sickness judgement; Clouser, Culver, and Gert’s account would engender positive disease and sickness judgements only for PA subgroups with significantly increased risk of harm, and negative judgements otherwise; Wakefield’s and Cooper’s accounts would probably find both judgements negative; and the social (risk of) harm account would find both judgements positive. Finally, with respect to the U group: on the BST, Wakefield’s harmful dysfunction account, and Clouser, Culver, and Gert’s account, both judgements would be negative; on Cooper’s account, they would depend on the presence of harmful symptoms; and on the social (risk of) harm account, they would be straightforwardly positive.

**Table 1:** Summary of different accounts of disease

	PS	PA	N	U
**BST**				
DJ	Yes	Yes	No	No
SJ	Probably yes (depending on social values)	Probably no (depending on social values)	No	No
**HD**				
DJ	Yes	Probably no	No	No
SJ	Yes	Probably no	No	No
**CCG**				
DJ	Yes	Yes/No (depending on the group considered)	No	No
SJ	Yes	Yes/No (depending on the group considered)	No	No
**CT**				
DJ	Yes (with exceptions)	No (with exceptions)	No	Yes/No (depending on symptoms
SJ	Yes (with exceptions)	No (with exceptions)	No	Yes/No (depending on symptoms)
**SH**				
DJ	Yes	Yes	No	Yes
SJ	Yes	Yes	No	Yes

BST: Boorse’s biostatistical theory; CT: Cooper’s tripartite account; CCG: Clouser, Culver, and Gert’s account; DJ: disease judgement; HD: Wakefield’s harmful dysfunction account; N: negative group; PA: positive and asymptomatic group; PS: positive and symptomatic group; SH: social (risk of) harm account; SJ: sickness judgement; U: untested group

## Concepts of disease and pandemics

In their responses to the COVID-19 pandemic, many governments have attributed the sick role to people who are asymptomatic and even to those who are untested or negative.^11^ Such pervasive positive sickness judgements are apparent in countries that have imposed strong lockdowns and quarantines for their whole population in order to suppress the outbreak of the virus, as Italy, Spain, and China have over different periods.^12^ On the one hand, with some variation from country to country, lockdown and quarantine periods have seen the extension of sickness exemptions and benefits (subject to employment status), including paid sick leave, tax credits, health insurance support, emergency cash transfers, stimulus payments, food vouchers, utility waivers, and loan forgiveness [53, 56, 57]. On the other hand, these privileges have coincided with the imposition of social obligations, suspension of certain rights, and restriction of usual activities not just for people who test positive but for the population writ large. Such measures have included stay-at-home orders (confining people to their place of residence except as necessary to perform work in essential critical infrastructure sectors), kindergarten and school closures, and domestic and international travel restrictions. Implicitly, positive sickness judgements have been extended to all citizens as a precaution, regardless of whether a given individual has been tested for the infection. Evidence for this is found in the widespread obligation to wear protective masks and gloves in public places, or even just outside one’s residence, as though everyone were contagious. Such measures may be considered only mildly intrusive in some respects, as they are generally only lightly enforced by local authorities, but very intrusive in others, as most people subjected to them have been neither infected nor exposed. However, because measures related to COVID-19 are motivated by community-wide risk and apply to entire populations, how much risk one person has or poses to others is of little relevance [58]. There are ethical reasons to support governments’ use of a heavy-handed quarantine approach in emergency situations [59].

As seen in the previous section, under all of the accounts of disease we present, a positive sickness judgement is taken to presuppose a positive disease judgement. Therefore, it can be said that the current COVID-19 pandemic in many countries exhibits not only pervasive positive sickness judgements, but also pervasive positive disease judgements. Moreover, as mentioned in the first section, we are assuming that disease judgements map to disease concepts such that it is possible to evaluate which disease concept is the most descriptively adequate to the present situation.

To that end, considering the accounts of disease reviewed above, the concept that best fits the pattern of disease and sickness judgements observed during the COVID-19 pandemic is the social (risk of) harm account, which involves social harm or social risk of harm as a constituent (i.e., a disease is a condition that is usually associated with some harm, or increased risk of harm, either to the subject or to someone other than the subject). This concept of disease—unlike the others—straightforwardly allows a positive sickness judgement for both the PA and the U groups. According to our matrix, however, it would not allow a positive sickness judgement for the N group.^13^ Still, it is reasonable to posit that the social (risk of) harm account of disease is the one that best describes the disease and sickness judgements evinced in the actual practices brought to bear in many countries during the COVID-19 pandemic.

In general, concepts can be reflected on with either a conservative or a revisionary attitude. The conservative strategy consists in identifying the concept that describes a given phenomenon and endorsing it (e.g., Boorse describes and endorses a pathological concept of disease). The revisionary strategy proceeds by identifying the concept that describes a given phenomenon and then challenging or criticizing it. Here we take the latter path, in a cautionary form. We point to three possible drawbacks of endorsing a concept of disease as social (risk of) harm—if such a concept were to be uncritically endorsed beyond the context of a public health emergency. In fact, should the social (risk of) harm concept of disease be extended outside specific exigent and contingent circumstances, it would have consequences that are distinctly undesirable, at least from our point of view.

First, under the social (risk of) harm definition of disease, a condition that is harmful or potentially harmful for society can be considered a disease irrespective of its physiological basis. As a result, the scientific and biological aspect of disease would be overshadowed, with the effect that certain types of perceived social deviance, such as homosexuality and drapetomania, or behavioural proclivities, such as heavy drinking, could easily come to be regarded as diseases in their own right.

Second, in endorsing the social (risk of) harm definition of disease, disease and sickness judgements could run counter to illness judgements—being, as we note above, judgements by which someone is recognized and described as suffering from or distressed by a condition. Of course, illness judgements may diverge under other disease concepts too, as long as the disease definition eschews individual harm as a criterion (as the BST does). Still, the subjective and phenomenological aspect of disease would be overshadowed, as asymptomatic people would count as diseased simply because they represent a risk to society. As a result, the patient’s first-person perspective would likely be entirely ignored.

Third, given that the social (risk of) harm account of disease would regard not only the PS group but also the PA and U groups as diseased, the number of diseased people would be seen to dramatically increase, which may create a problem of overdiagnosis. In the specific case of COVID-19, overdiagnosis may in fact be beneficial, both for individuals and for society—for instance, it may help to contain local outbreaks or alert governments so that they may appropriately allocate additional health care resources, such as hospital beds, doctors, and available treatments. Still, overdiagnosis is not just a theoretical problem in which many people would count as diseased without biological basis and irrespective of their first-person experience; it is also a practical problem that may have harmful effects if allowed to persist beyond the state of emergency—such as causing individual psychological distress and anxiety, increasing health care costs, and creating issues for health care fairness (as resources would be inappropriately allocated and therefore ideally subtracted from those more entitled to benefit from them) [60].

There are two possible replies to our argument. First, one could say that concepts of disease are local and situated, and in the context of a pandemic, a suitable concept of disease may be temporarily adopted for use within a specific time and place. In other words, a pluralist view of disease concepts could be advanced [61, 62]. Second, one could object that a concept of disease as social (risk of) harm does not accurately describe practices in a pandemic because these practices—sick role attribution, in particular—are not medical, but political. Specifically, one could say that when people who are asymptomatic or untested are judged to be sick, those judgements are rooted in considerations that are political, rather than medical, and so remain neutral about which concept of disease is being employed. For example, the pandemic situation might actually be well accounted for by a naturalist point of view, just with the added stipulation that people who are asymptomatic, negative, or untested may be judged to be sick in the absence of any pathological condition or symptom if there are political or prudential reasons for judging them so. In this way, disease and sickness judgements would be totally detached.

We acknowledge both of these objections. The first is correct in proposing that a conceptual pluralism about disease is at least logically coherent. However, a thorough discussion of how pluralism can function across contexts and medical specialties would far exceed the scope of this paper [63]. The second objection is also correct insofar as it can be argued that it is always true that more than one concept can describe a practice adequately, as demonstrated convincingly by Ludwig Wittgenstein in his rule-following paradox [64, 65]. Thus, it may well be the case that in the event of a pandemic, medical concepts of disease are overruled by political concepts of disease. Here, however, the discussion would move away from the philosophy of medicine and would need to expand on the relationship between medical experts and political power.

To recap, in this section, we have claimed that the concept of disease that best explains the disease and sickness judgements observed during the COVID-19 pandemic and is the most descriptively adequate to the present situation is that of disease as social (risk of) harm; alternatively, sickness judgements could be totally divorced from disease judgements and instead rendered based on purely political or prudential considerations. Moreover, we suggest that extending the social (risk of) harm account of disease beyond the emergency circumstances of the pandemic could have undesirable consequences. In so doing, however, we have endeavoured neither to offer a positive concept of disease for use by authorities during a pandemic nor to criticize the policies and measures that have actually been implemented by various governments.

## Conclusion

In this paper, we showed how different philosophical accounts of disease have implications not only for what counts as a disease and, by extension, for who may be regarded as having a disease (disease judgements), but also for who may be accorded the privileges and responsibilities that society assigns to people who are sick (sickness judgements). To that end, we ran a simple thought experiment considering an ideal diagnostic test for an infectious disease such as COVID-19 with regard to four groups of people—positive and symptomatic; positive and asymptomatic; negative; untested. We observed that on all the accounts of disease considered, the positive and symptomatic group and the negative group would receive positive and negative disease judgements, respectively, with sickness judgements patterning in kind. However, only the social (risk of) harm account of disease would yield straightforward positive disease and sickness judgements both for people in the positive and asymptomatic group and for people in the untested group.

In keeping with these observations, we noted that the social (risk of) harm account of disease is the one that best describes the actual response measures implemented by many countries during the COVID-19 pandemic. We then took a modest revisionary strategy, challenging the uncritical endorsement of a social (risk of) harm concept of disease, especially outside the context of an emergency. In fact, we think that such an account would overshadow not only the scientific and biological aspect of disease, but also the subjective and phenomenological aspect. On the one hand, people could count as diseased even in the absence of a dysfunction; on the other hand, people could count as diseased even without experiencing any current or potential harm. As a corollary, overdiagnosis would dramatically increase.

Finally, we acknowledged the possibility of endorsing pluralism in the concept of disease or admitting that during a pandemic the concept of disease is shaped by political and not theoretical considerations. Even so, such assumptions should be made explicit by authorities in order to avoid the risks that would follow from uncritically endorsing an account of disease as social (risk of) harm beyond the current state of emergency.

## Footnotes

1 In what follows, we use the term ‘disease’ in a very broad sense to encompass all pathological conditions [6–9]. Sometimes, other terms, such as ‘malady’ [10, 11] or ‘disorder’ [12, 13], are used in this sense as well.

2 A false positive for a diagnostic test is someone who tests positive but does not have the condition being tested, while a false negative is someone who tests negative but does in fact has the condition. The specificity of a test measures its ability to avoid false positives, while the sensitivity of a test measures its ability to avoid false negatives. Accuracy refers to the overall weighted average of these two measurements.

3 In so doing, we do not claim to offer a philological and thorough interpretation of the theories of disease we examine, but merely use them as general examples of how the concept of disease can be understood.

4 To be precise, Wakefield aims to define the notion of disorder, but for our purposes this can be equated with disease.

5 To be clear, the dysfunction requirement is conceived in very different terms by Boorse and Wakefield.

6 Clouser et al. actually set out to define the notion of malady, but again, for our purposes this can be equated with disease.

7 According to Clouser et al., a distinct sustaining cause is ‘a cause whose effects come and go simultaneously (or nearly so) with its respective presence and absence’ [10, p. 32].

8 Clouser et al. actually talk about ‘basic evil’ rather than ‘basic harm’ [10].

9 The *DSM* does not define dysfunction. Of course, this is a problem (as we argue elsewhere [49]), but it cannot be addressed here. Moreover, it is not relevant for our purposes to establish what the exact meaning of dysfunction is.

10 We bracket off the dysfunction requirement in order to focus on a definition that is totally centered on harm, or risk of harm, either to the subject or to someone else. This may make for an awkward philosophical definition of disease, but, as we discuss in the next section, it seems to reflect the current situation presented by the COVID-19 pandemic.

11 A negative test, however, may mitigate some restrictions—for instance, in some situations negative people may not have to quarantine or may be allowed to travel by plane.

12 There are various reports on the lockdown measures in effect in various countries at different times [52–55]. These reports show how such measures extend not only to those who are positive and symptomatic but also to others, thus implicitly endorsing a social (risk of) harm concept of disease. It is worth noting, however, that in this paper we are making a claim that is not sociological but conceptual, which still holds in a conditional form: if such-and-such policies are implemented, then the concept of disease behind them would be such-and-such. Of course, we believe that our evidential basis is correct, and we do not intend our claim to be read in the conditional form; nevertheless, reinforcement of the empirical evidence is not required for our line of argument to obtain.

13 Assuming the social (risk of) harm account of disease, however, it might be argued that in some contexts—such as during a pandemic—the mere fact of being in public would seem to satisfy the requirements for positive disease and sickness judgments. Owing to the epistemic limits of diagnostic testing and a relative lack of understanding about the mechanisms of disease spreading, being in public—even with a negative test result—might increase the risk of individual exposure to the virus as well as the chance of spreading it to other people and thereby harming them. This state of affairs would ostensibly point to a *potentiated* social (risk of) harm concept of disease. In such case, our analysis would be strengthened, as a potentiated social (risk of) harm account of disease would wholly describe the disease and sickness judgements observed during the COVID-19 pandemic. We thank Katelyn MacDougald for making us reflect on this point.
